# Meloxicam ameliorates the systemic inflammatory response syndrome associated with experimentally induced endotoxemia in adult donkeys

**DOI:** 10.1111/jvim.15783

**Published:** 2020-05-28

**Authors:** Francisco Javier Mendoza Garcia, Carlos Gonzalez‐De Cara, Raul Aguilera‐Aguilera, Antonio Buzon‐Cuevas, Alejandro Perez‐Ecija

**Affiliations:** ^1^ Department of Animal Medicine and Surgery University of Cordoba, Campus Rabanales, Road Madrid‐Cadiz km 396, 14104 Cordoba Spain; ^2^ Egabro Veterinary Practice Cabra, Cordoba Spain

**Keywords:** equids, interleukins, SIRS, selective COX‐2 inhibitors, sepsis

## Abstract

**Background:**

Little information is available about endotoxemia in donkeys. Characterizing the systemic inflammatory response (SIRS) to lipopolysaccharide (LPS) in donkeys would provide valuable clinical and therapeutic information. The effects of meloxicam on endotoxemia have not been studied in this species.

**Objectives:**

To study the pathophysiology and gene expression associated with experimentally induced endotoxemia, and evaluate the effects of meloxicam on experimentally induced endotoxemia in donkeys and in equine monocyte cultures.

**Animals:**

Six healthy adult female donkeys.

**Methods:**

Endotoxemia was induced by an IV infusion of LPS for 30 minutes. Animals either received 20 mL of saline or 0.6 mg/kg of meloxicam IV after LPS infusion. The experiments lasted 6 hours. Blood samples were collected serially for hematology, serum biochemistry, interleukin measurement, and leukocyte gene expression analysis. Vital signs were recorded throughout the study. Monocyte cultures were used to test the effects of meloxicam on LPS‐activated monocytes.

**Results:**

Lipopolysaccharide induced fever, leukopenia, and neutropenia of similar magnitude in both groups, but meloxicam attenuated increases in plasma lactate, tumor necrosis factor‐alpha (TNFα), and interleukin 1β concentrations compared to controls. No differences were detected between groups for cytokine mRNA expression. Furthermore, meloxicam decreased TNFα release in LPS‐activated monocyte cultures.

**Conclusions and Clinical Importance:**

Meloxicam could be a feasible option for the treatment of endotoxemia and SIRS in donkeys. Additional studies are necessary to investigate possible meloxicam‐related posttranscriptional regulation and to compare this drug with other nonsteroidal anti‐inflammatory drugs (NSAIDs) in animals with endotoxemia.

**Abbreviations**
CFT: cutaneous fold time retraction
COX‐2: cyclooxygenase 2
CRT: capillary refill time
DP: digital pulse
GAPDH: glyceraldehyde 3‐phosphate dehydrogenase
Hgb: hemoglobin concentration
HR: heart rate
IL‐1β: interleukin 1β
IL‐6: interleukin 6
IL‐8: interleukin 8
IL‐10: interleukin 10
LD: left dorsal colon
LV: left ventral colon
MCH: mean corpuscular hemoglobin
MCHC: mean corpuscular hemoglobin concentration
MCV: mean corpuscular volume
MMC: mucous membrane color
MPV: mean platelet volume
NSAIDs: nonsteroidal anti‐inflammatory drugs
PCT: plateletcrit
PDW: platelet distribution width
PLT: platelet count
RBC: red blood cell count
RD: right dorsal colon
RDW: red cell distribution width
RR: respiratory rate
RT: rectal temperature
RV: right ventral colon
TL: toxic line
TNFα: tumor necrosis factor‐alpha
WBC: white blood cell count

## 1 INTRODUCTION

The donkey is a species of emerging interest in veterinary practice, and is responsible for an increasing number of veterinary consultations and visits to referral hospitals.^1^ Numerous anatomical, genetic, endocrine, and metabolic differences have been identified between donkeys and horses,^2^, ^3^, ^4^, ^5^, ^6^, ^7^ and extrapolating information from horses to donkeys can lead to diagnostic errors and unnecessary treatments, with consequent health risks and economic impact.^8^

Endotoxemia is assumed to be as common in donkeys as in horses, and is mostly associated with disorders such as enterocolitis, neonatal septicemia, strangulating colic, and other conditions.^9^, ^10^ Although endotoxemia has been researched extensively in horses,^11^, ^12^, ^13^, ^14^ little information is available on this condition in donkeys.^15^ The extent of the systemic inflammatory response syndrome (SIRS) in response to lipopolysaccharide (LPS) shows marked interspecies variability,^16^, ^17^ and species‐specific research will generate valuable information with pathophysiologic, clinical, and therapeutic implications. Moreover, the inherent stoic behavior of donkeys requires detailed characterization of the clinical signs that appear during endotoxemia in order to better recognize this condition in donkeys.^18^

Several therapeutic options have been tested in endotoxemic horses, including Toll‐like receptor 4 (TLR‐4) antagonists, LPS blockers (eg, polymyxin B, antibodies), anti‐inflammatory drugs (eg, flunixin meglumine, eltenac, pentoxifylline, ethyl pyruvate), local and systemic anesthetic drugs (eg, lidocaine, ketamine),^19^, ^20^, ^21^, ^22^, ^23^ with nonsteroidal anti‐inflammatory drugs (NSAIDs), specifically flunixin meglumine,^24^ being the most commonly used treatments. Because of the adverse effects of nonselective NSAIDs on mucosal healing, dorsal colon health, and renal blood flow,^24^ selective cyclooxygenase 2 (COX‐2) inhibitors, such as meloxicam or firocoxib, could be a feasible and safer option for the treatment of endotoxemia in donkeys.

Our objectives were to study the pathophysiology and gene expression of experimentally induced endotoxemia in donkeys and evaluate the effects of meloxicam on systemic, hematological, gene expression, and in vitro variables in healthy donkeys with experimentally induced endotoxemia.

## 2 MATERIALS AND METHODS

### 2.1 Animals

Six healthy adult (7.6 ± 0.8 years old) nonpregnant female Andalusian donkeys, weighing 348.3 ± 38.9 kg, maintained on the premises of the Veterinary Teaching Hospital were used. Donkeys were deemed healthy based on medical history, physical examination, and hematological and biochemical analyses. No medication had been administered in the 6 months before the study, and the animals had no previous history of endotoxemia‐inducing disease (eg, colic, pleuropneumonia, diarrhea).

The study received approval from the Welfare Committee of Animal Experimentation of the University of Cordoba (2015PI/05, approval date: March 19th, 2015) and the Rural Development, Fishing and Agriculture Ministry of Junta de Andalucia (19‐03‐2015‐212, approval date: March 19, 2015). Animals were handled according to national guidelines for research animals.

### 2.2 Experimental design

Donkeys were housed in box stalls 1 day before commencement of the experiment, and polyurethane catheters (Milacath, Mila International Inc., Kentucky) were placed aseptically in both jugular veins and the lines were irrigated with 5 mL sterile saline every 4 hours. Experiments were performed after 12 hours of fasting the animals (ie, food was withheld from 8 pm the night before the experiment and water from 1 hour before).

Endotoxemia was induced as previously described in horses.^20^, ^25^, ^26^, ^27^, ^28^, ^29^ Briefly, an LPS (*Escherichia coli* O55:B5, Sigma‐Aldrich Quimica, Madrid, Spain) dose of 20 ng/kg was administered in 500 mL sterile saline by infusion over 30 minutes (designated −30‐0 minutes) using a volumetric infusion pump (Infusomat, Braun VetCare, Barcelona, Spain) into the left jugular vein. Blood samples for hematology, biochemistry, interleukin, and gene expression analysis were collected from the right jugular catheter.

Donkeys were randomly assigned to receive either a single IV bolus of 20 mL saline (control group) or meloxicam 0.6 mg/kg (Loxicom, Norbrook, Northern Ireland, United Kingdom; meloxicam group) after LPS infusion (designated 0 minutes post‐LPS infusion [PLI]). The animals were interchanged between groups after a 1 month washout period, and thus 2 trials were carried out on each animal. Systemic inflammatory response syndrome was considered to be present when at least 2 of the following criteria were met: tachycardia, tachypnea, fever, or abnormal white blood cell count.^30^

The following clinical variables were measured every 15 minutes from −30 minutes (before LPS infusion) until 240 minutes PLI: heart and respiratory rates (HR and RR, respectively), rectal temperature (RT), capillary refill time (CRT), mucous membrane color (MMC), toxic line (TL) presence, cutaneous fold time retraction (CFT), 4‐quadrant gut motility (RD, right dorsal colon; LD, left dorsal colon; RV, right ventral colon; LV, left ventral colon), and digital pulse (DP). An additional physical examination was performed at 360 minutes PLI.

### 2.3 Hematology, biochemistry, and plasma and in vitro interleukin determination

Blood for automated hematology analysis (Lasercyte, Idexx Laboratories SL, Hoofddorp, The Netherlands) was collected into K_3_‐EDTA tubes (Becton Dickinson, Plymouth, United Kingdom) at −30, 0, 30, 60, 90, 120, 150, 180, 240, and 360 minutes PLI. Manual packed cell volume (PCV) was determined and the following variables were measured: white blood cell count (WBC), differential leukocyte counts, red blood cell count (RBC), hemoglobin concentration (Hgb), mean corpuscular volume (MCV), mean corpuscular hemoglobin (MCH), mean corpuscular hemoglobin concentration (MCHC), red cell distribution width (RDW), platelet counts (PLT), mean platelet volume (MPV), plateletcrit (PCT), and platelet distribution width (PDW).

Total solids and plasma fibrinogen concentrations were determined in lithium‐heparin plasma (Becton Dickinson, Eysins, Switzerland) at the aforementioned time points by refractometry and heat denaturation methods, respectively.

Blood for determinations of plasma glucose and lactate concentrations was collected into sodium fluoride tubes (Becton Dickinson, Eysins, Switzerland) and measured by spectrophotometry (A15 Biosystems, Barcelona, Spain) at −30, 0, 30, 60, 90, 120, 180, and 240 minutes PLI. Tubes were centrifuged 10 minutes at 1200*g* and plasma kept at −20°C until measurements were made.

Plasma tumor necrosis factor‐alpha (Equine TNFα ELISA Reagent Kit, ThermoScientific, Massachusetts) and interleukin 1β (Equine IL‐1β VetSet, Kingfisher Biotech Inc., Minnesota) concentrations were determined using equine‐validated ELISA kits,^28^, ^31^, ^32^ at −30, 0, 30, 60, 90, 120, 180, and 240 minutes PLI. Blood samples were centrifuged for 10 minutes at 1200*g* and plasma was kept at −20°C until measurements were carried out.

### 2.4 In vitro monocyte cultures

Before LPS infusion, 80 mL of blood was collected aseptically into K_3_‐EDTA tubes by jugular puncture. Peripheral blood mononuclear cells were isolated by modifying reported protocols used for horses.^33^, ^34^ Briefly, blood mixed with an equal volume of Hanks' balanced salt solution (HBSS, Sigma‐Aldrich, St. Louis, Missouri) was layered over Ficoll‐Paque Plus 1073 (Sigma‐Aldrich) and centrifuged at 900*g* for 30 minutes. Cells were washed and resuspended in 20 mL Roswell Park Memorial Institute 1640 (RPMI‐1640) medium with l‐glutamine, penicillin, streptomycin, and amphotericin B (antibiotic antimycotic solution, Sigma‐Aldrich) and 10% equine serum (Sigma‐Aldrich). An aliquot was counted on a hemocytometer and >95% viability was confirmed by trypan blue (Sigma‐Aldrich) exclusion assay.

Mononuclear cells were plated onto 150 × 20 mm sterile tissue culture plates at a concentration of 1 × 10^7^ cells/dish and incubated at 37°C in a 5% CO_2_ atmosphere for 2 hours. Nonadherent cells were removed using warm media, and the remaining cells were detached by incubation with chilled 10 mM EDTA. This protocol for the isolation of viable monocytes (>85%) has been verified previously using Cytospin (ThermoScientific, Massachusetts) slides stained with trypan blue and a nonspecific esterase stain.^35^

Triplicate sterile plates containing 5 × 10^6^ monocytes/dish were prepared with 1 of the following treatments: equine media only (control group), media containing 100 pg/mL of LPS (*E. coli* O55:B5, Sigma‐Aldrich Quimica, Madrid, Spain), media with meloxicam at 1 μg/mL (Loxicom, Norbrook, Northern Ireland, United Kingdom), or media containing 100 pg/mL of LPS and 1 μg/mL of meloxicam. After incubation for 6 hours at 37°C in 5% CO_2_, the cell culture medium was removed, centrifuged for 10 minutes at 800*g* and stored at −80°C until cytokine measurement. The LPS dose for in vitro experiments was chosen based on previous experiments in horses.^21^, ^34^ The meloxicam concentration was chosen based on the reported mean serum concentration in donkeys 1 hour after administration.^36^

### 2.5 Quantitative leukocyte gene expression

Blood samples were collected into Tempus RNA blood tubes (ThermoFisher Scientific, Darmstadt, Germany) at the following time points: −30, 0, 30, 60, 90, and 180 minutes PLI, and kept at −80°C until RNA extraction.

Ribonucleic acid was extracted using the Tempus Spin RNA Isolation kit (ThermoFisher Scientific, Darmstadt, Germany) and stored at −80°C. Quantification of RNA was performed using ultraviolet‐visible spectrophotometry (NanoDrop ND‐1000, NanoDrop Technologies, Wilminton, Delaware), and RNA quality was determined using TapeStation (Agilent Technologies, Palo Alto, California). Complementary DNA was synthesized from 500 ng RNA in a Veriti Thermocycler (Applied Biosystems, California). Quantitative PCR (qPCR) was performed using a LightCycler 480 (Roche, Basel, Switzerland). Samples were amplified in triplicate. Glyceraldehyde 3‐phosphate dehydrogenase (GAPHD) was used as a control housekeeping gene in qPCR analyses. The results were analyzed using LightCycler 480 software 1.5.0 (Roche, Basel, Switzerland).

Characteristics and sequences of primers for TNFα, interleukins 1β, 6, 8, and 10, and glyceraldehyde 3‐phosphate dehydrogenase (GAPDH) are shown in Table 1. Previously reported horse gene sequences were compared (nucleotide BLAST, NCBI resources, Rockville Pike, Bethesda, Maryland) with the most recent *Equus asinus asinus* assembly (identification number: ASM303372v1).^37^, ^38^, ^39^, ^40^ Specific primers for donkeys were designed for TNFα (only forward primer), IL‐6,IL‐8,IL‐10, and GAPDH using the primer designing tool Primer‐BLAST (NCBI resources). Primers were tested in silico (NCBI resources) to detect any product abnormality, and PCR amplicon sequences were verified by Sanger sequencing (Big Dye Terminator V3.1, 3730XL, Applied Biosystems, California).

**TABLE 1:** Sequences and properties of primers for TNFα, interleukins 1β, 6, 8, and 10, and GAPDH genes in donkeys

Gene	Primers sequences (5′ ‐ 3′)	Length (b)	Tm (°C)	GC (%)	Product length (bp)	Reference	Horse accession number	Horse homology (%)
TNFα	f: AAGTGACAAGCCTGTAGCCC	20	63.2	55	272	37 ^a^	NM_001081819.2	99
	r: TCTTGATGGCAGAGAGGAGGTTGAC	25	70.6	52				
IL‐1β	f: TGTACCTGTCTTGTGGGACGAAA	23	67.5	47.8	185	39	XM_001495926.5	88
	r: TTCTGCTTGAGAGGTGCTGA	20	64.0	50				
IL‐6	f: CACCACTGGTCTTTCGGAGT	20	64.2	55	163	^a^	NM_001082496.2	100
	r: AGTTGGGTCAGGGGTGGTTA	20	65.4	55				
IL‐8	f: TTACTGCAGAGCTTCGGTGC	20	65.5	55	165	^a^	NM_001083951.2	98
	r: TTGTATGGGGGTTCAGGCAG	20	67.0	55				
IL‐10	f: CTAGGGAACGAAGCATCCAGG	21	66.8	57.1	134	^a^	NM_001082490.1	99
	r: TCAGGAGAGAGGTACCACAGG	21	63.3	57.1				
GAPDH	f: ATTGCCCTCAACGACCACTT	20	65.7	50	140	^a^	NM_001163856.1	99
	r: TCTTGCTGGGTGATTGGTGG	20	68.4	55				

Abbreviations: b, base; Bp, base pair; °C, celsius degree; f, forward; GAPDH, glyceraldehyde 3‐phosphate dehydrogenase; GC, guanine‐cytosine content; IL, interleukin; r, reverse; Tm, melting temperature; TNFα, tumor necrosis factor‐alpha.

a New primers designed for donkeys.

### 2.6 Data analysis

Normality was assessed using the Kolmogorov‐Smirnov test. Normally distributed data are expressed as the mean and SD, and an analysis of variance (ANOVA) of repeated measures followed by a Fisher's least significant difference (LSD) post hoc analysis was carried out to determine differences between repeated measures. Data that were not normally distributed (CRT, MMC, TL, CFT, gut motility, and DP) are expressed as median and interquartile range (IQR, 25th‐75th percentile), and significance was determined using a Friedman test followed by a Wilcoxon test. Differences between groups at each sampling point were determined using either a Mann‐Whitney or *t* test depending on data distribution. Percentiles were calculated using the Tukey's‐Hinges test. Correlations were determined using a Pearson or Spearman's test depending on data distribution. A *P* value <.05 was considered significant. Statistical analysis was performed using commercial statistical software (IBM SPSS Statistics 24, IBM, Chicago, Illinois).

Categorical variables were measured by the same clinician to avoid intra‐ and inter‐assay bias. Mucous membrane color was classified as: 0, pale; 1, pink; 2, congested; or 3, cyanotic. Toxic line and DP were classified as: 0, normal; or 1, increased. Gut motility was grouped as: 0, absent; 1, decreased; 2, normal; or 3, increased.

## 3 RESULTS

### 3.1 Effect of LPS on clinical signs in control and meloxicam groups

**FIGURE 1:**
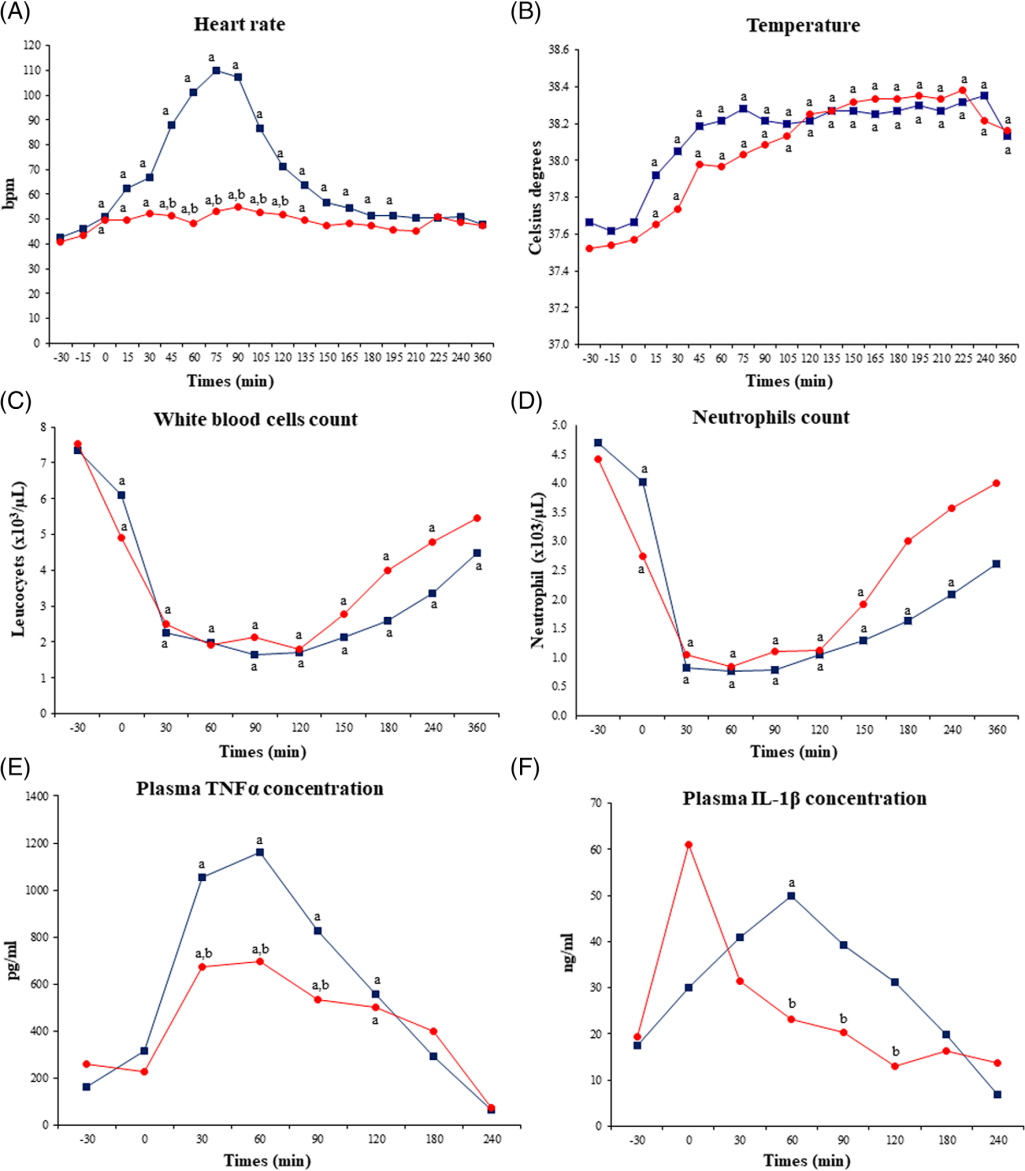
Clinical variables (A and B), total white blood cell (C) and neutrophil (D) counts, and plasma TNFα (E) and IL‐1β (F) concentrations in donkeys (n = 6) receiving LPS and either a single IV bolus of saline (20 mL) or IV meloxicam (0.6 mg/kg). Data are expressed as mean. Blue line (squares) represents the control group, and red line (circles) represents the meloxicam group. ^a^ *P* < .05 versus −30 minutes; ^b^ *P *< .05 versus similar time‐point between groups. IL, interleukin; LPS, lipopolysaccharide; TNFα, tumor necrosis factor‐alpha

All donkeys met the SIRS criteria and safely completed the study. Lipopolysaccharide induced a significant (*P* < .05) HR increase from 0 to 195 minutes PLI in the control group, peaking at 75 minutes, whereas meloxicam attenuated this increase (Figure 1A). Significant differences were observed between groups from 45 to 120 minutes PLI (Figure 1A).

Although tachypnea was observed from 15 to 90 minutes in the control group (Table S1), RR was within reference range from 90 minutes up until the end of the experiment. Meloxicam ameliorated the effect of LPS infusion on RR.

Lipopolysaccharide infusion led to a significant (*P* < .05) increase in body temperature in both groups. Temperature increased more slowly in the meloxicam group, peaking at 150 minutes PLI, compared to the control group in which temperature peaked at 75 minutes PLI (Figure 1B).

The CRT was significantly (*P* < .05) prolonged (from 75 minutes PLI to the end of the experiment) and mucous membranes were significantly (*P* < .05) congested (from 75 to 135 minutes) in both groups (Table S1). Presence of TL was observed and apparent until the end of the experiment in both groups, but it appeared significantly (*P* < .05) later in meloxicam‐treated donkeys (90 minutes) compared with the control group (30 minutes; Table S1).

Gut motility was unchanged in the meloxicam group, whereas it was significantly (*P* < .05) decreased between 45 and 120 minutes PLI in the control group, with no difference in decrease among abdominal quadrants (Table S1).

Digital pulse was not augmented and CFT was not prolonged in any group.

### 3.2 Hematological and biochemical changes induced by LPS

Lipopolysaccharide induced leukopenia and neutropenia from 0 to 360 minutes PLI (Figure 1C,D), as well as lymphopenia, monocytopenia and eosinopenia from 30 to 360 minutes PLI in both groups (*P* < .05, Table 2). No differences were observed between groups.

**TABLE 2:** Hematological variables following administration of saline or meloxicam to experimentally induced endotoxemic donkeys

Variable	Group	−30 (min)	0 (min)	30 (min)	60 (min)	90 (min)	120 (min)	150 (min)	180 (min)	240 (min)	360 (min)
PCV (%)	LPS + saline	37.3 ± 4.6	34.7 ± 6.7	37.7 ± 6.1	38.7 ± 6.3	40.1 ± 7.4	38.3 ± 5.0	38.2 ± 4.6	39.5 ± 5.0^a^	41.1 ± 6.1^a^	41.5 ± 6.5^a^
	LPS + meloxicam	34.5 ± 5.1	33.8 ± 3.6	32.9 ± 2.4^b^	33.2 ± 3.1	33.7 ± 1.4^b^	33.0 ± 3.2^b^	34.3 ± 2.7	34.7 ± 3.1	35.2 ± 3.3^b^	38.0 ± 3.7^b^
RBC (×10^6^/μL)	LPS + saline	7.0 ± 1.1	7.2 ± 1.1	7.4 ± 1.0^a^	7.3 ± 1.3	7.4 ± 1.2^a^	7.2 ± .9	7.1 ± .9	7.1 ± .9	7.3 ± 1.1	7.5 ± 1.0^a^
	LPS + meloxicam	6.6 ± 1.0	6.5 ± .9	6.2 ± .7	6.2 ± .9	6.3 ± 1.0	6.3 ± 1.1	6.4 ± 1.0	6.3 ± .9	6.4 ± 1.0	6.4 ± 1.0
Hgb (g/dL)	LPS + saline	14.2 ± 2.0	13.9 ± 1.6	14.1 ± 2.3	14.2 ± 2.5	14.8 ± 2.5	14.4 ± 2.2	14.6 ± 2.2	14.8 ± 1.8^a^	14.8 ± 2.0^a^	14.8 ± 2.4^a^
	LPS + meloxicam	13.3 ± 1.3	13.0 ± 1.2	12.7 ± 1.2	12.8 ± 1.4	12.8 ± .8^b^	13.1 ± 1.0^b^	13.1 ± 1.5^b^	13.1 ± 1.5	13.1 ± 1.1	13.3 ± .9^b^
MCV (fL)	LPS + saline	55.3 ± 2.8	54.3 ± 2.7	54.6 ± 2.2	54.9 ± 2.2	55.6 ± 2.4	55.8 ± 2.5	55.3 ± 1.7	55.1 ± 2.3	55.2 ± 1.9	56.1 ± 2.8
	LPS + meloxicam	54.9 ± 2.6	54.5 ± 2.0	56.0 ± 1.7	55.7 ± 2.0	55.6 ± 2.2	55.0 ± 1.9	54.7 ± 1.4	55.4 ± 2.2	55.1 ± 1.9	54.7 ± 2.1
MCH (pg)	LPS + saline	20.2 ± 1.1	19.4 ± 1.6	18.9 ± 1.6	19.6 ± 2.8	19.9 ± 1.3	19.9 ± 1.6	20.7 ± 1.9	20.8 ± 1.4	20.2 ± 2.2	19.7 ± 1.8
	LPS + meloxicam	20.4 ± 2.5	20.1 ± 2.0	20.8 ± 2.3	20.8 ± 2.6	20.7 ± 2.5	21.3 ± 3.1	20.8 ± 2.1	21.0 ± 1.5	20.6 ± 2.4	20.9 ± 2.8
MCHC (g/dL)	LPS + saline	38.2 ± 1.3	37.7 ± 2.6	36.0 ± 1.0^a^	37.0 ± 2.0	37.3 ± 1.4	37.1 ± 1.6^a^	37.9 ± 2.2	37.5 ± 1.3	36.6 ± .7^a^	36.6 ± 1.5^a^
	LPS + meloxicam	38.5 ± 2.2	38.0 ± 1.6	37.6 ± 1.4	38.1 ± 1.7	38.1 ± 1.7	38.7 ± 2.7	38.3 ± 2.4	37.7 ± 1.3	37.2 ± 2.1	37.4 ± 1.1
RDW (%)	LPS + saline	19.1 ± .5	19.2 ± .5	19.1 ± .4	18.9 ± .5	19.1 ± .6	18.1 ± .3^a^	18.3 ± .4^a^	18.3 ± .7^a^	18.2 ± .2^a^	18.5 ± .6^a^
	LPS + meloxicam	19.3 ± .5	18.9 ± .2^a^	18.9 ± .4^a^	19.1 ± .4	19.0 ± .4^a^	18.7 ± .7^a^	19.0 ± 1.1	19.1 ± .2	18.8 ± .6	18.7 ± .5^a^
PLT (×10^3^/μL)	LPS + saline	211.0 ± 37.4	205.5 ± 79.0	187.5 ± 42.5	202.5 ± 53.9	181.3 ± 30.0	207.3 ± 23.0	203.8 ± 22.0	203.8 ± 35.4	196.0 ± 45.4	217.0 ± 58.6
	LPS + meloxicam	246.0 ± 36.4	233.3 ± 38.7	226.3 ± 48.4	220.3 ± 25.5	201.2 ± 29.5	214.7 ± 33.4	210.7 ± 25.8	228.2 ± 46.3	224.0 ± 37.0	223.2 ± 32.5
MPV (fL)	LPS + saline	6.3 ± 1.1	6.7 ± .7	6.4 ± 1.9	6.1 ± 1.0	6.1 ± 1.1	6.8 ± 1.2	6.7 ± 1.2	6.7 ± 1.2	6.7 ± .8	6.4 ± .4
	LPS + meloxicam	6.1 ± .7	6.4 ± .6	6.4 ± .7	6.5 ± .8	6.4 ± .4	6.3 ± .4	6.4 ± .6	6.2 ± .3	6.4 ± .4	6.2 ± .5
PCT (%)	LPS + saline	.13 ± .03	.14 ± .04	.10 ± .07	0.10 ± .03^a^	.09 ± .03^a^	.12 ± .02	.11 ± .02^a^	.12 ± .03	.13 ± .04	.14 ± .05
	LPS + meloxicam	.15 ± .02	.12 ± .03^a^	.14 ± .02	0.14 ± .03	.12 ± .05	.13 ± .04	.14 ± .03^a^	.15 ± .03	.14 ± .03	.14 ± .02
PDW (%)	LPS + saline	21.4 ± 1.7	20.9 ± 1.5	21.2 ± 2.0	19.3 ± .5^a^	20.2 ± 1.1	19.7 ± .7^a^	20.3 ± 1.0	19.6 ± .7	19.6 ± .7	19.7 ± .8
	LPS + meloxicam	20.0 ± 1.0	20.6 ± 2.7	20.5 ± .5^b^	19.4 ± 1.1^b^	19.7 ± 1.4	19.2 ± 1.2	19.2 ± 1.0	20.1 ± 1.3	19.3 ± 1.2	19.7 ± 1.5
Lym (×10^3^/μL)	LPS + saline	1.8 ± .5	1.5 ± .3	1.1 ± .1^a^	1.0 ± .5	.6 ± .2^a^	.4 ± .1^a^	.6 ± .1^a^	.7 ± .2^a^	.9 ± .3^a^	1.4 ± .8
	LPS + meloxicam	2.1 ± 1.1	1.5 ± .6^a^	1.1 ± .6^a^ ^,^ ^b^	.9 ± .5^a^	.8 ± .4^a^	.5 ± .3^a^	.6 ± .2^a^	.6 ± .3^a^	.8 ± .3	.9 ± .3^a^
Mono (×10^3^/μL)	LPS + saline	.4 ± .1	.3 ± .1^a^	.1 ± .03^a^	.1 ± .02^a^	.05 ± .01^a^	.05 ± .04^a^	.1 ± .05^a^	.1 ± .05^a^	.1 ± .1^a^	.2 ± .1^a^
	LPS + meloxicam	.4 ± .1	.2 ± .1^a^	.1 ± .02^a^	.1 ± .05^a^	.1 ± .02^a^ ^,^ ^b^	.1 ± .03^a^	.1 ± .03^a^	.1 ± .1^a^	.2 ± .1^a^	.2 ± .06^a^
Eos (×10^3^/μL)	LPS + saline	.4 ± .3	.3 ± .2	.2 ± .1^a^	.1 ± .2^a^	.1 ± .1^a^	.1 ± .2^a^	.2 ± .2	.2 ± .1^a^	.2 ± .2^a^	.3 ± .1
	LPS + meloxicam	.6 ± .6	.4 ± .4^a^	.2 ± .1^a^	.1 ± .1^a^	.1 ± .1^a^	.1 ± .1^a^	.2 ± .1^a^	.2 ± .1^a^	.3 ± .2^a^	.3 ± .3^a^

*Note:* Data are expressed as mean ± SD.

Abbreviations: Eos, eosinophils; Hgb, hemoglobin concentration; LPS, lipopolysaccharide; Lym, lymphocytes; MCV, mean corpuscular volume; MCH, mean corpuscular hemoglobin; MCHC, mean corpuscular hemoglobin concentration; MPV, mean platelet volume; Mono, monocytes; PCV, packed cell volume; PCT, plateletcrit; PDW, platelet distribution width; PLT, platelet count; RBC, red blood cells; RDW, red cell distribution width.

a *P* < .05 versus −30 min.

b *P* < .05 versus control at the same time‐point.

Significant differences (*P* < .05) were observed in PCV, RBC, and hemoglobin concentration between groups. Additional hematological differences are presented in Table 2.

**FIGURE 2:**
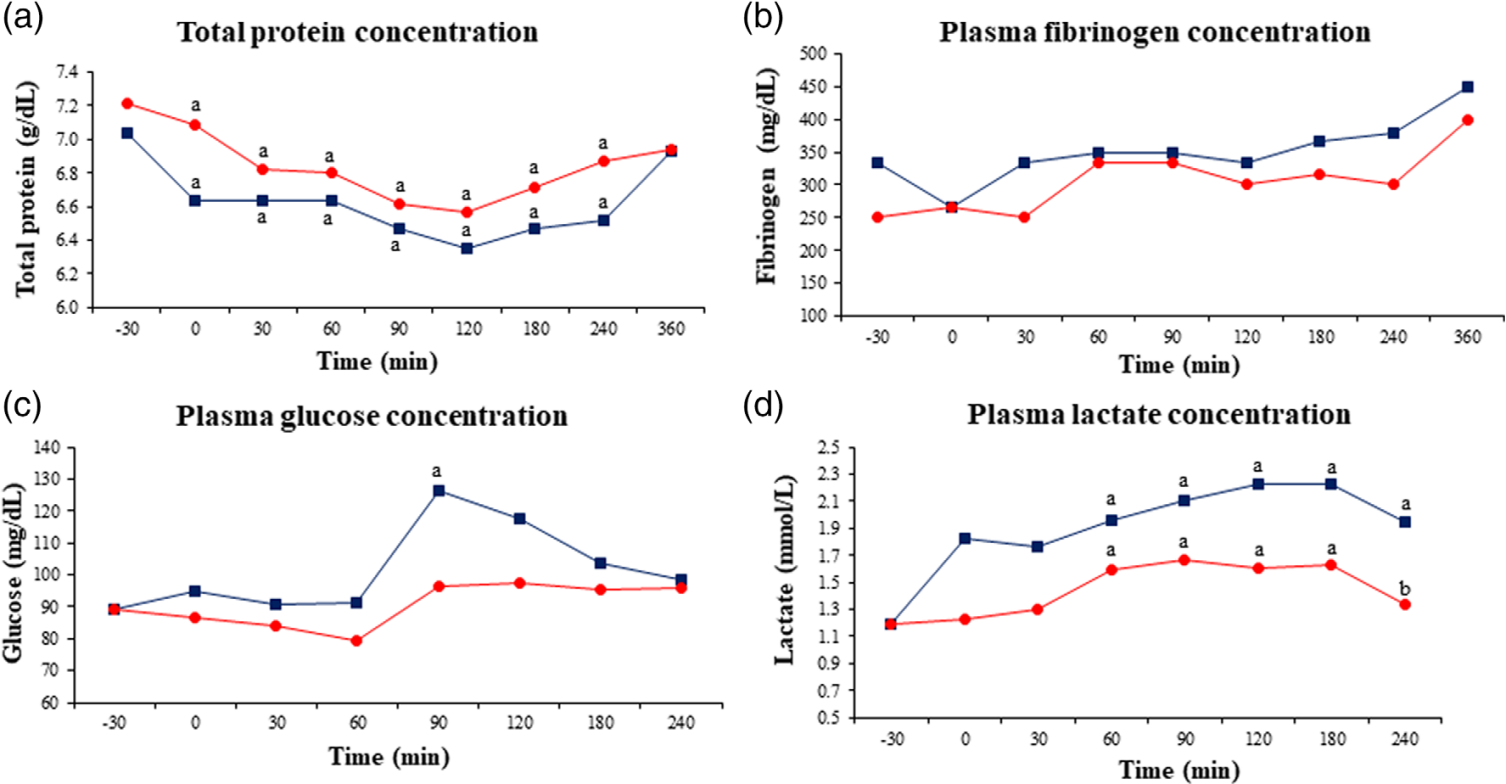
Biochemical variables in experimentally induced endotoxemic donkeys (n = 6) after administration of either a single IV bolus of saline (20 mL) or IV meloxicam (0.6 mg/kg). Data are expressed as mean. Blue line (squares) represents the control group, and red line (circles) represents the meloxicam group. ^a^ *P* < .05 versus −30 minutes; ^b^ *P* < .05 versus similar time‐point between groups

A significant (*P* < .05) decrease in plasma total solids concentration was observed between 0 and 240 minutes in both groups (Figure 2A). Plasma fibrinogen concentration did not change in either group (Figure 2B). No differences between groups were detected in these variables.

Although an increase in plasma glucose concentration was detected at 90 minutes in both groups (higher in the control group), no differences were observed between groups (Figure 2C).

Lipopolysaccharide infusion induced a significant (*P* < .05) increase in plasma lactate concentration in both groups, but meloxicam attenuated this increase, resulting in a more rapid return to baseline (240 minutes) compared to the control group (Figure 2D).

### 3.3 Plasma and in vitro interleukin concentrations

**FIGURE 3:**
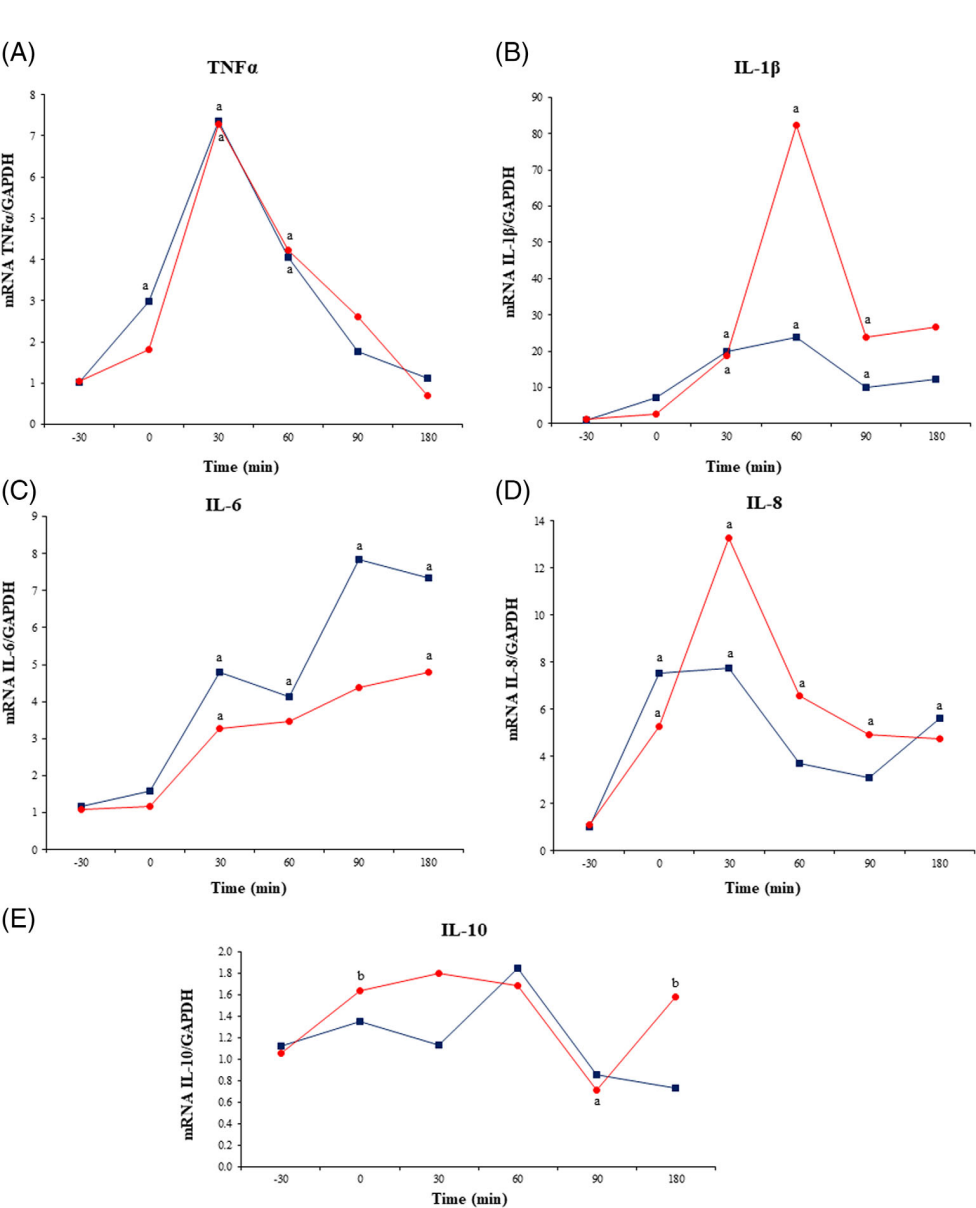
Quantitative leukocyte mRNA expression of TNFα (A), IL‐1β (B), IL‐6 (C), IL‐8 (D), and IL‐10 (E) in experimentally‐induced endotoxemic donkeys (n = 6) after administration of either a single IV bolus of saline (20 mL) or IV meloxicam (0.6 mg/kg). Gene expression was corrected for GAPDH mRNA expression. Blue line (squares) represents the control group, and red line represents the meloxicam group. ^a^ *P* < .05 vs −30 minutes

Plasma TNFα concentration increased significantly (*P* < .05) in both groups from 30 to 120 minutes PLI (Figure 1E), but this increase was significantly less in meloxicam‐treated donkeys compared to controls (Figure 1E). Lipopolysaccharide also induced an increase in TNFα concentration in vitro, and this increase was attenuated by meloxicam (Figure 3A).

Lipopolysaccharide infusion led to an increase in plasma IL‐1β concentration (Figure 1F) in both groups, but plasma IL‐1β concentrations returned to baseline faster in meloxicam‐treated donkeys compared to the control group (60 versus 180 minutes PLI, respectively; *P* < .05). Although no statistical differences were observed between groups, in vitro IL‐1β concentrations were higher in the LPS group compared to the other groups (Figure 3B).

Correlations between plasma interleukins concentrations and other variables are compiled in Tables S2 and S3.

### 3.4 Quantitative analysis of leukocyte gene expression

**FIGURE 4:**
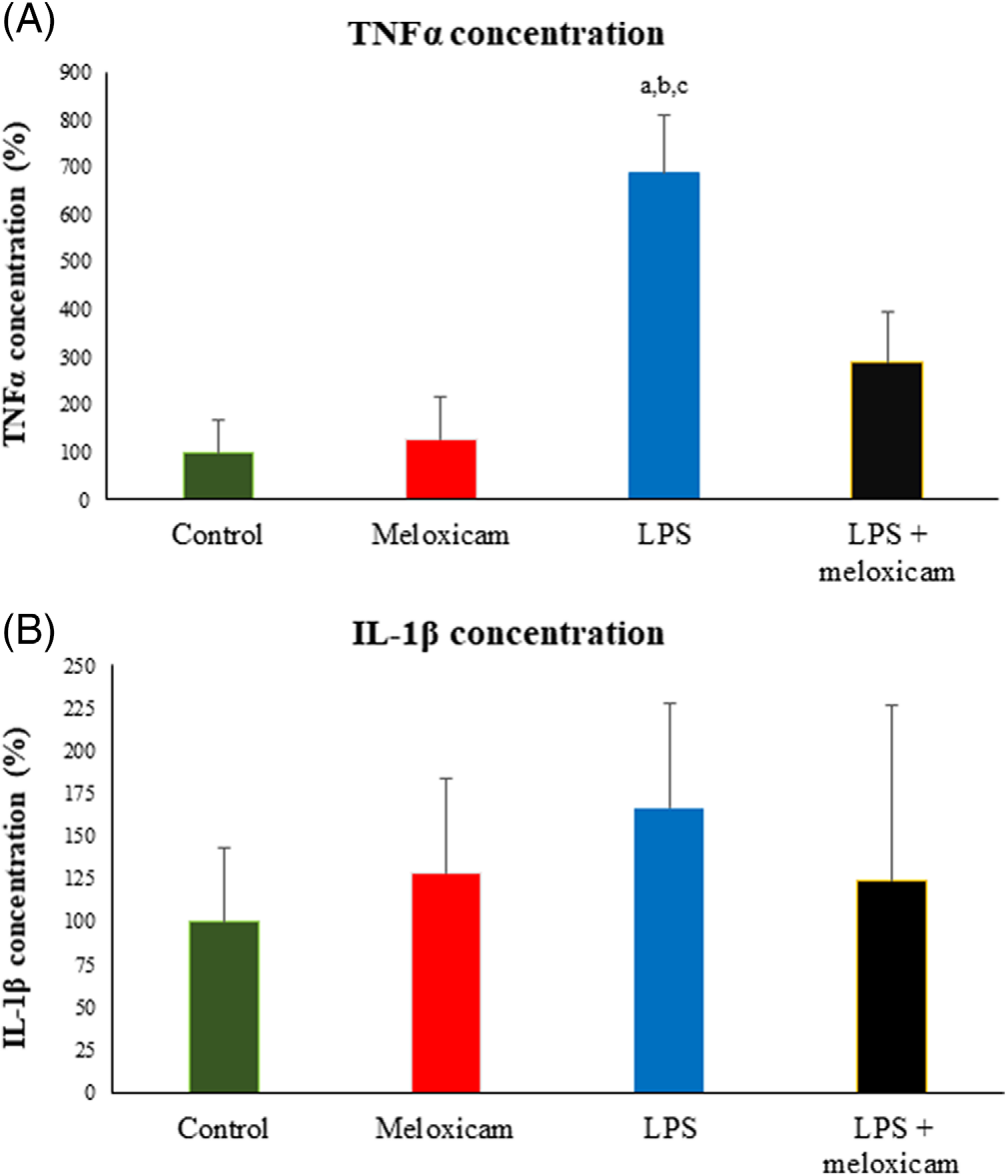
TNFα (A) and IL‐1ß (B) concentrations in *in vitro* monocyte cultures supernatants. Data are expressed as percentages of the control group. ^a^ *P* < .01 vs control; ^b^ *P* < .05 vs meloxicam; ^c^ *P* < .05 vs LPS+meloxicam

Lipopolysaccharide infusion resulted in an increase in pro‐inflammatory interleukins TNFα, IL‐1β, IL‐6, and IL‐8 gene expression in both groups (Figure 4). Although TNFα expression returned to baseline at 90 minutes in both groups, IL‐1β, IL‐6, and IL‐8 expression remained increased throughout the experiment. No statistical differences were observed between groups. Significant differences (*P* < .05) in IL‐10 gene expression were observed between groups, with a more rapid increase (peaking at 30 minutes and returning to baseline at 90 minutes, *P* < .05) in meloxicam‐treated donkeys compared to the control group (Figure 4E).

Correlations between gene expression and other variables are presented in Tables S2 and S3.

## 4 DISCUSSION

We characterized the effect of meloxicam in healthy adult donkeys after LPS challenge. Lipopolysaccharide infusion induced tachycardia, fever, gut hypomotility, a severe decrease in WBC (including neutrophils) counts, and an increase in serum concentrations of lactate and TNFα. In addition, total leukocyte mRNA expression of TNFα, IL‐1β, IL‐6, and IL‐8 was increased. Treatment with meloxicam attenuated many of the harmful effects of LPS. In addition, meloxicam attenuated the increase in TNFα caused by LPS in monocyte cultures.

Lipopolysaccharide administration caused marked tachycardia, although mean HR was noticeably higher compared to previous reports in horses.^20^, ^23^, ^25^, ^26^, ^29^ Meloxicam administration significantly decreased and delayed the onset of tachycardia in donkeys. In contrast, although administered PO, meloxicam administration did not have this effect in horses with induced endotoxemia.^41^

In our study, LPS induced marked fever in donkeys, as described in horses,^20^, ^23^, ^25^ but a previous study in donkeys failed to detect fever until 3 hours PLI.^15^ Meloxicam did not prevent fever in donkeys, similar to that observed in horses treated PO.^41^

Gut motility was significantly decreased after LPS administration in donkeys, as has been observed in horses,^11^, ^42^ whereas phenylbutazone pretreatment in endotoxemic horses decreased this harmful effect.^43^ Ours is the first study to show the effect of meloxicam on gut motility in equids.

Leukopenia with neutropenia, lymphopenia, eosinopenia, and monocytopenia was observed at 0 minutes PLI in both groups, which was earlier compared to other studies (1 hour PLI).^12^, ^15^, ^20^, ^23^, ^26^, ^27^ The meloxicam group returned to baseline more rapidly than did the control group. This finding is in agreement with reports in horses after PO administration of meloxicam or other NSAIDs, such as flunixin meglumine.^11^, ^41^

In donkeys, the LPS‐induced decrease in plasma total solids was observed sooner compared to horses.^22^, ^23^ Variations in plasma fibrinogen concentrations were not significant between groups or compared to baseline, as has been observed in horses,^23^ suggesting late onset of this acute phase protein in donkeys, and equids in general.

Flunixin meglumine has been shown to alleviate the increase in plasma lactate concentration in endotoxemic horses,^11^ and herein we describe a similar effect of meloxicam in donkeys.

An increase in TNFα (plasma concentrations and RNA expression) after LPS infusion was observed sooner in donkeys compared to horses.^17^, ^20^, ^23^, ^27^, ^29^, ^40^, ^41^, ^44^ This finding could explain the rapid onset of clinical signs such as tachycardia and leukopenia in donkeys. Nonetheless, the time it took for these 2 variables to return to baseline in donkeys was similar to that reported for horses.^17^, ^23^, ^27^, ^29^, ^41^, ^44^ Meloxicam treatment significantly decreased plasma TNFα concentrations, contrary to findings in horses after PO administration of meloxicam,^41^ or other drugs such as flunixin meglumine, eltenac, ketamine, lidocaine, or ethyl pyruvate.^22^, ^23^, ^34^ Meloxicam treatment did not alter TNFα mRNA expression, and therefore the discrepancy observed between plasma TNFα concentration and mRNA expression could be attributed to posttranscriptional changes.^45^

The time frame of IL‐1β increased mRNA expression was similar to that reported for horses.^17^, ^29^ No data are available concerning plasma IL‐1β concentrations in horses. The discrepancy in IL‐1β mRNA expression and plasma concentrations in the meloxicam group also could be explained by a translational effect.^46^

Similar to horses, expression of IL‐6, IL‐8, and IL‐10 mRNA increased PLI in donkeys.^17^, ^29^, ^40^, ^44^ The effect of meloxicam on mRNA expression of these cytokines has not been studied in horses.

Correlations between cytokine mRNA expression and clinical variables were in agreement with previous findings in horses,^22^, ^44^, ^45^ highlighting the importance of these mediators in the response to LPS in donkeys.

In vitro results for TNFα matched those seen in vivo and are similar to those seen in horses,^14^, ^31^ with meloxicam preventing this increase. On the other hand, the discrepancies observed between in vivo and in vitro data on the effect of meloxicam on IL‐1β could be a consequence of factors related to measurement in supernatants or to lack of a complete leukocyte population in these cultures. No data are available to date on the in vitro effects of meloxicam in horses.

Based on our results, meloxicam could be of interest for clinicians treating donkeys with endotoxemia and SIRS, because of its ability to control clinical variables associated with this syndrome while avoiding adverse effects such as nephrotoxicity or intestinal damage observed with nonselective NSAIDs.

One limitation of our study is the possible presence of endotoxin tolerance. Tolerance to LPS is defined as a 75% decrease in plasma TNFα concentrations after a repeat endotoxin exposure.^16^, ^31^ In our study, no differences in plasma TNFα concentrations were observed in donkeys between trials, and because the effects of other cytokines or pathogen‐associated molecular pattern (PAMP) sensors (eg, TLR‐4) were not evaluated, endotoxin tolerance therefore cannot be excluded. Moreover, interindividual variation in LPS response occurs, and possible subclinical LPS exposure cannot be excluded. Finally, although a dose of 20 ng/kg LPS clearly induced typical clinical signs and responses associated with endotoxemia, a higher dose or a longer experimental time frame might have identified additional differences between groups, or novel effects of meloxicam.

## 5 CONCLUSIONS

Ours is the first study to characterize the effects of meloxicam in LPS‐challenged adult donkeys, both in vivo and in vitro. Additional studies are necessary to investigate possible meloxicam‐related posttranscriptional regulation and in vivo cytokine interactions causing the clinical features, as well as studies directly comparing meloxicam and other NSAIDs in donkeys with endotoxemia.

## CONFLICT OF INTEREST DECLARATION

Authors declare no conflict of interest.

## OFF‐LABEL ANTIMICROBIAL DECLARATION

Authors declare no off‐label use of antimicrobials.

## INSTITUTIONAL ANIMAL CARE AND USE COMMITTEE (IACUC) OR OTHER APPROVAL DECLARATION

Our study received approval from both the Welfare Committee of Animal Experimentation of the University of Cordoba (2015PI/05, approval date: March 19th, 2015) and the Rural Development, Fishing and Agriculture Ministry of Junta de Andalucia (19‐03‐2015‐212, approval date: March 19th, 2015). Animals handled according to national guidelines for research animals.

## HUMAN ETHICS APPROVAL DECLARATION

Authors declare human ethics approval was not needed for this study.

## Supporting information


**Table S1** Supporting Information.

**Table S2** Supporting Information.

**Table S3** Supporting Information.
