# Supplementary material for: Meloxicam ameliorates the systemic inflammatory response syndrome associated with experimentally induced endotoxemia in adult donkeys
Source: J Vet Intern Med. 2020 May 28;34(4):1631–41. doi: 10.1111/jvim.15783 (PMC7379049; doi:10.1111/jvim.15783)
Supplement: Supplementary file 1 — Table S1 Supporting Information. [file JVIM-34-1631-s001.pdf]

Physical variables in donkeys receiving intravenous saline or meloxicam after endotoxemia induction with LPS.

Variable	Group	-30 (min)	-15 (min)	0 (min)	15 (min)	30 (min)	45 (min)	60 (min)	75 (min)	90 (min)	105 (min)	120 (min)	135 (min)	150 (min)	165 (min)	180 (min)	195 (min)	210 (min)	225 (min)	240 (min)	360 (min)
MMC	LPS+ saline	1 (0)	1 (0)	1 (.25)	1 (0)	1 (.13)	1 (.13)	1.25 (.63)	1.5 (1.25)	1.5 (1)[a]	1.5 (1)[a]	1.5 (.63)[a]	1.5 (.63)[a]	1 (.5)	1 (.13)	1 (.13)	1 (.13)	1 (.13)	1 (.13)	1 (0)	1 (.13)
	LPS+ meloxi	1 (0)	1 (0)	1 (.13)	1 (.13)	1 (.13)	1 (.75)	1 (.50)	1.25 (.50)[a]	1.25 (.50)[a]	1.5 (.50)[a]	1.5 (.50)[a]	1.25 (1)	1 (1.13)	1 (.63)	1 (.75)	1 (.13)	1 (0)	1 (.13)	1 (.13)	1 (.25)
CRT (sec)	LPS+ saline	1 (0)	1 (0)	1 (0)	1 (0)	1 (0)	1 (0)	1 (1)	2 (1.5)[a]	2 (.5)[a]	2 (.75)[a]	2 (1)[a]	2 (.5)[a]	2 (.5)[a]	2 (1)[a]	2 (1)[a]	2 (1.25)[a]	2 (1)[a]	1.5 (1.25)[a]	2 (1.25)[a]	1.5 (1.25)[a]
	LPS+ meloxi	1 (0)	1 (0)	1 (0)	1 (0)	1 (.38)	1.5 (1.75)	1 (1.25)	2 (1.25)[a]	2 (1.63)[a]	2.5 (2)[a]	3 (1.25)[a]	3 (1.5)[a]	2.5 (1.38)[a]	2.25 (1.5)[a]	2 (2.25)[a]	2.25 (1.75)[a]	2 (1.63)[a]	2 (1.25)[a]	2 (1)[a]	2 (1.25)[a]
TL	LPS+ saline	0 (0)	0 (0)	0 (0)	0 (0)	0 (1)	0 (1)	1 (.25)[a]	1 (.25)[a]	1 (.25)[a]	1 (.25)[a]	1 (.25)[a]	1 (.25)[a]	1 (.25)[a]	1 (.25)[a]	1 (.25)[a]	1 (1)[a]	1 (1)[a]	1 (1)[a]	1 (1)[a]	1 (1)[a]
	LPS+ meloxi	0 (0)	0 (0)	0 (0)	0 (0)	0 (0)	0 (0)	0 (0)[b]	0 (0)[b]	0.5 (1)[a]	1 (1)[a]	1 (.63)[a]	1 (.63)[a]	1 (.63)[a]	0.75 (1)[a]	1 (1)[a]	1 (1)[a]	1 (1)[a]	1 (1)[a]	1 (.63)[a]	1 (.75)[a]
CFT (sec)	LPS+ saline	1 (0)	1 (0)	1 (0)	1 (0)	1 (0)	1 (0)	1 (0)	1 (0)	1 (0)	1 (0)	1 (0)	1 (0)	1 (0)	1 (0)	1 (0)	1 (0)	1 (0)	1 (0)	1 (0)	1 (0)
	LPS+ meloxi	1 (0)	1 (0)	1 (0)	1 (0)	1 (0)	1 (0)	1 (0)	1 (0)	1 (0)	1 (0)	1 (0)	1 (0)	1 (0)	1 (0)	1 (0)	1 (0)	1 (0)	1 (0)	1 (0)	1 (0)
DP	LPS+ saline	0 (0)	0 (0)	0 (0)	0 (0)	0 (0)	0 (0)	0 (0)	0 (0)	0 (0)	0 (0)	0 (0)	0 (0)	0 (0)	0 (0)	0 (0)	0 (0)	0 (0)	0 (0)	0 (0)	0 (0)
	LPS+ meloxi	0 (0)	0 (0)	0 (0)	0 (0)	0 (0)	0 (0)	0 (0)	0 (0)	0 (0)	0 (0)	0 (0)	0 (0)	0 (0)	0 (0)	0 (0)	0 (0)	0 (0)	0 (0)	0 (0)	0 (0)
RD	LPS+ saline	2 (0)	2 (0)	2 (0)	2 (0)	2 (0)	1.5 (.5)	1 (.25)[a]	1.5 (.5)[a]	1.5 (.5)[a]	1.5 (.5)[a]	1 (1)[a]	2 (0)	2 (0)	2 (0)	2 (0)	2 (0)	2 (0)	2 (0)	2 (0)	2 (0)
	LPS+ meloxi	2 (0)	2 (0)	2 (0)	2 (.5)	2 (.5)	2 (1.5)	2 (1.25)	2 (.25)	2 (.25)	2 (1)	2 (1)	2 (1.25)	2 (1.5)	2 (0)	2 (.5)	2 (.5)	2 (.5)	2 (.5)	2 (.5)	2 (1)
RV	LPS+ saline	2 (0)	2 (0)	2 (0)	2 (0)	2 (0)	1.5 (.5)	1 (1)[a]	1 (1)[a]	1.5 (.5)[a]	2 (0)[a]	1.5 (.5)[a]	2 (0)	2 (0)	2 (0)	2 (0)	2 (0)	2 (0)	2 (0)	2 (0)	2 (0)
	LPS+ meloxi	2 (0)	2 (0)	2 (0)	2 (0)	2 (.5)	2 (1.5)	2 (1)	2 (.25)	2 (.25)	2 (1.25)	2 (1)	2 (1)	2 (.75)	2 (.25)	2 (.5)	2 (.5)	2 (.5)	2 (.5)	2 (.5)	2 (1)
LD	LPS+ saline	2 (0)	2 (0)	2 (0)	2 (0)	1.5 (.5)	1.5 (.5)[a]	1 (1)[a]	1.5 (.5)[a]	1 (1)[a]	1.5 (.5)[a]	1.5 (.5)[a]	2 (0)	2 (0)	2 (0)	2 (0)	2 (0)	2 (0)	2 (0)	2 (0)	2 (0)
	LPS+ meloxi	2 (0)	2 (0)	2 (0)	2 (.5)	2 (.5)	2 (1.5)	2 (1)	2 (.25)	2 (1)	2 (1)	2 (1)	2 (1)	2 (.5)	2 (.25)	2 (.5)	2 (.5)	2 (.25)	2 (.25)	2 (.5)	2 (1)
LV	LPS+ saline	2 (0)	2 (0)	2 (0)	2 (0)	2 (.5)	1.5 (.5)[a]	1 (1)[a]	1 (1)[a]	1 (1)[a]	1.5 (.5)[a]	1.5 (.5)[a]	2 (0)	2 (0)	2 (0)	2 (0)	2 (0)	2 (0)	2 (0)	2 (0)	2 (0)
	LPS+ meloxi	2 (0)	2 (0)	2 (0)	2 (0)	2 (.5)	2 (1)	2 (1.25)	2 (1.25)	2 (1)	2 (1)	2 (1)	2 (1)	2 (.75)	2 (.25)	2 (.5)	2 (.5)	2 (.5)	2 (.5)	2 (.5)	2 (1)
RR (rpm)	LPS+ saline	23.2 ±8.0	24.0 ±7.3	23.8 ±6.4	26.4 ±4.0	23.8 ±4.8	23.0 ±3.4	25.6 ±2.7	26.4 ±3.5	29.4 ±4.8	22.4 ±4.7	21.2 ±3.1	22.0 ±4.6	19.6 ±5.3	19.8 ±5.7	18.8 ±5.2	20.4 ±5.4	19.0 ±4.6	19.2 ±5.7	21.0 ±8.4	20.8 ±5.0
	LPS+ meloxi	28.0 ±11.8	25.0 ±4.3	23.6 ±2.9	21.8 ±2.0	21.7 ±2.4	20.5 ±4.6	20.8 ±3.3	20.0 ±2.8	22.0 ±3.1	21.3 ±2.7	21.2 ±2.4	20.8 ±2.0	20.2 ±2.3	21.2 ±1.3[b]	20.7 ±2.1[b]	20.4 ±2.1[b]	19.4 ±.9[a,b]	18.7 ±2.2[a,b]	21.2 ±1.1[a]	20.0 ±1.6[a]

MMC: mucous membrane color; CRT: capillary refill time; TL: toxic line; CFT: cutaneous fold retraction time; DP: digital pulse; RD: right dorsal motility; RV: right ventral motility; LD: left dorsal motility; LV: left ventral motility; RR: respiratory rate. Data with normal distribution are expressed as mean ± standard deviation, and those with no normal distribution as median and (interquartile range). Mucous membrane color was classified as: 0, pale; 1, pink; 2, congested; and 3, cyanotic. Toxic line and digital pulse were classified as: 0, absence; and 1: presence. Gut motility was grouped as: 0, absence; 1, decreased; 2, normal; and 3, increased. Digital pulse was categorized as: 0, normal; and 1, increased. $^{a}P<.05$ vs -30 minutes; $^{b}P<.05$ vs control at the same time-point.
