# Supplementary material for: Meloxicam ameliorates the systemic inflammatory response syndrome associated with experimentally induced endotoxemia in adult donkeys
Source: J Vet Intern Med. 2020 May 28;34(4):1631–41. doi: 10.1111/jvim.15783 (PMC7379049; doi:10.1111/jvim.15783)
Supplement: Supplementary file 2 — Table S2 Supporting Information. [file JVIM-34-1631-s002.pdf]

Pearson correlations among main variables in the control group.

	Temperature	*WBC*	*TNFα*	*IL-1β*	*TNFα mRNA*	*IL-1β mRNA*	*IL-6mRNA*	*IL-8mRNA*	*IL-10mRNA*
HR	r=.33 p<.01	r=-.19 p=.05	r=.73 p<.01	r=.10 p=.38	r=.35 p=.01	r=.52 p<.01	r=.48 p<.01	r=-.02 p=.89	r=.15 p=.30
Temperature	-	r=-.23 p=.01	r=.26 p=.02	r=.17 p=.09	r=.29 p=.04	r=.31 p=.03	r=.31 p=.03	r=.08 p=.58	r=-.07 p=.62
WBC	-	-	r=-.46 p<.01	r=-.11 p=.28	r=-.01 p=.93	r=-.41 p<.01	r=-.57 p<.01	r=-.16 p=.26	r=.26 p=.07
TNFα	-	-	-	r=-.02 p=.85	r=.48 p<.01	r=.58 p<.01	r=.68 p<.01	r=.07 p=.66	r=-.13 p=.39
IL-1β	-	-	-	-	r=.49 p<.01	r=.16 p=.26	r=-.16 p=.27	r=-.05 p=.74	r=-.25 p=.09
TNFα mRNA	-	-	-	-	-	r=.59 p<.01	r=.03 p=.85	r=.35 p=.02	r=.03 p=.83
IL-1β mRNA	-	-	-	-	-	-	r=.28 p=.05	r=.41 p<.01	r=.12 p=.38
IL-6mRNA	-	-	-	-	-	-	-	r=.04 p=.77	r=-.17 p=.24
IL-8mRNA	-	-	-	-	-	-	-	-	r=.84 p<.01

HR: heart rate; WBC: white blood cells counts; r: Pearson coefficient; p: P value.
