# Supplementary material for: Meloxicam ameliorates the systemic inflammatory response syndrome associated with experimentally induced endotoxemia in adult donkeys
Source: J Vet Intern Med. 2020 May 28;34(4):1631–41. doi: 10.1111/jvim.15783 (PMC7379049; doi:10.1111/jvim.15783)
Supplement: Supplementary file 3 — Table S3 Supporting Information. [file JVIM-34-1631-s003.pdf]

Pearson correlations among main variables in the meloxicam group.

	Temperature	*WBC*	*TNFα*	*IL-1β*	*TNFα mRNA*	*IL-1β mRNA*	*IL-6mRNA*	*IL-8mRNA*	*IL-10mRNA*
HR	r=.45 p<.01	r=-.18 p=.06	r=.24 p=.02	r=.15 p=.14	r=.12 p=.41	r=.25 p=.09	r=.21 p=.15	r=.20 p=.18	r=.24 p=.10
Temperature	-	r=-.20 p=.04	r=.51 p<.01	r=-.17 p=.10	r=.08 p=.61	r=.40 p<.01	r=.58 p<.01	r=.24 p=.11	r=.17 p=.26
WBC	-	-	r=-.32 p<.01	r=.17 p=.09	r=-.40 p<01	r=-.24 p=.10	r=-.11 p=.45	r=-.30 p=.04	r=-.14 p=.34
TNFα	-	-	-	r=.05 p=.64	r=.64 p<.01	r=.61 p<.01	r=.64 p<.01	r=.64 p<.01	r=.09 p=.54
IL-1β	-	-	-	-	r=.32 p=.03	r=.21 p=.15	r=.04 p=.76	r=.46 p<.01	r=-.07 p=.65
TNFα mRNA	-	-	-	-	-	r=.41 p<.01	r=.27 p=.06	r=.81 p<.01	r=.15 p=.32
IL-1β mRNA	-	-	-	-	-	-	r=.59 p<.01	r=.40 p<.01	r=.08 p=.57
IL-6mRNA	-	-	-	-	-	-	-	r=.46 p<.01	r=-.12 p=.40
IL-8mRNA	-	-	-	-	-	-	-	-	r=.10 p=.50

HR: heart rate; WBC: white blood cells counts; r: Pearson coefficient; p: P value.
